# Exposure to cisplatin in the operating room during hyperthermic intrathoracic chemotherapy

**DOI:** 10.1007/s00420-021-01738-3

**Published:** 2021-06-30

**Authors:** Till Markowiak, Michael Ried, Christopher Larisch, Dennis Nowak, Hans-Stefan Hofmann, Stefan Rakete

**Affiliations:** 1 Department of Thoracic Surgery, University Medical Center Regensburg, Regensburg, Germany; 2 Germany and Comprehensive Pneumology Center Munich, University Hospital, LMU Munich, Institute and Clinic for Occupational, Social and Environmental Medicine, Munich, Germany; 3 Comprehensive Pneumology Center Munich, German Center for Lung Research, Munich, Germany; 4 Department of Thoracic Surgery, Hospital Barmherzige Brüder, Regensburg, Germany

**Keywords:** Hyperthermic intrathoracic chemotherapy, HITOC, Cisplatin, Surface contamination, Occupational exposure

## Abstract

**Purpose:**

Hyperthermic intrathoracic chemotherapy (HITOC) is an additive, intraoperative treatment for selected malignant pleural tumors. To improve local tumor control, the thoracic cavity is perfused with a cisplatin-containing solution after surgical cytoreduction. Since cisplatin is probably carcinogenic to humans, potential contamination of surfaces and pathways of exposure should be systematically investigated to enable risk assessments for medical staff and thus derive specific recommendations for occupational safety.

**Methods:**

Wipe sampling was performed at pre-selected locations during and after ten HITOC procedures, including on the surgeon's gloves, for the quantitation of surface contaminations with cisplatin. After extraction of the samples with hydrochloric acid, platinum was determined as a marker for cisplatin by voltammetry.

**Results:**

High median concentrations of cytostatic drugs were detected on the surgeons’ (1.73 pg Cis-Pt/cm^2^, IQR: 9.36 pg Cis-Pt/cm^2^) and perfusionists’ (0.69 pg Cis-Pt/cm^2^, IQR: 1.73 pg Cis-Pt/cm^2^) gloves. The display of the perfusion device showed partially elevated levels of cisplatin up to 4.92 pg Cis-Pt/cm^2^ and thus could represent an origin of cross-contamination. In contrast, cisplatin levels on the floor surfaces in the area of the surgeon and the perfusion device or in the endobronchial tube were relatively low.

**Conclusion:**

With a correct use of personal protective equipment and careful handling, intraoperative HITOC appears to be safe to perform with a low risk of occupational exposure to cisplatin.

## Introduction

Hyperthermic intrathoracic chemotherapy (HITOC) is an innovative, additive treatment for selected patients with primary or secondary malignant pleural tumors. In Germany, about 350 of these procedures have been performed in 10 years by 17 specialized clinics, and the number is increasing (Ried et al. 2018). Over a period of 60 min, the thoracic cavity is perfused with a heated solution to improve local tumor control after surgical cytoreduction (Ried et al. 2013). There is no international standardized procedure for the intra- and perioperative care of HITOC patients (Ried et al. 2016; Zhou et al. 2017). However, the perfusion solution always contains cisplatin in a dosage between 50 and 250 mg/m^2^ body surface area (Maury et al. 2017; Zellos et al. 2009; Zhou et al. 2017). High local concentrations of cisplatin can be achieved in the lung tissue down to a median depth of 3–4 mm and thus induce cell death of residual tumor cells by the intracavitary cisplatin lavage (Ried et al. 2015; Cregan et al. 2013). The results on long-term survival are promising (Markowiak et al. 2019; Ambrogi et al. 2018). Cisplatin is probably carcinogenic for humans and, therefore, represents a potential risk for the healthcare personnel (Falck et al. 1979; International Agency for Research on Cancer; Pethran et al. 2003; Dranitsaris et al. 2005). For this reason, occupational exposure should always be avoided and a cautious handling and the use of protective equipment is mandatory.

Dermal uptake was identified to be the most likely route of occupational exposure to antineoplastic drugs in healthcare settings (Kromhout et al. 2000; Sessink et al. 1994). As a result, Pethran et al. demonstrated that platinum excretion of pharmacy employees preparing cytostatic agents is significantly higher during the working shift (Pethran et al. 2003). Also in the hospital setting an incorporation by administering employees could be observed, since it has been demonstrated that the urine of healthcare workers after occupational exposure to chemotherapeutic agents was mutagenic to indicator organisms like *Escherichia coli* and *Salmonella typhimurium* (Falck et al. 1979). Furthermore, a meta-analysis showed a 46% increase in the risk of spontaneous abortion for female medical personal after exposure to chemotherapeutic agents (Dranitsaris et al. 2005). These results urge caution, but dermal contact can be prevented by taking the appropriate safety measures (Landeck et al. 2015). To prevent the exposure of the operating room (OR) personnel, specific safety regulations must be applied (safety instruction, gloves compatible with cytostatic drugs, protective surgical gowns, safety glasses, dedicated containers for disposal of cytostatic agents) (Ried et al. 2018).

In this context, the intraoperative application of cytostatic drugs is a challenge for the surgeon, since this procedure requires high demands on the tightness of wound and drainage sutures. Leaking perfusion solution can represent a hazard for the personnel through direct or indirect contact with contaminated surfaces. Another specific aspect of HITOC is the close physical distance of the intrathoracic volume and the bronchial system. It is unclear if the patient's bronchial secretion contains cisplatin and, therefore, may lead to contamination of materials used in ventilation of the patient.

Since cytostatic drugs are only used in a few procedures in the environment of the OR, there are only few studies assessing a possible risk to the personnel in this regard. The aim of this study was the identification of vulnerable locations in the OR to prevent distribution of the substance. These findings can be used to verify adequate protective measures for the staff. In contrast to oncological wards, handling of cytostatic drugs is often unfamiliar to staff of an OR. This can result in distrust of the intraoperative HITOC. The objectives of this study was to evaluate the occupational safety of HITOC in terms of exposure to cisplatin for the medical staff and allow specific recommendations for the operative working environment.

## Methods

### Study design

This prospective observational study considered all patients who received HITOC after surgical cytoreduction between January 2020 and November 2020 in the University Hospital Regensburg and the Hospital of Barmherzige Brüder Regensburg. Patients with less than 60 min of intrathoracic perfusion or an intrathoracic dose of cisplatin other than 175 mg/m^2^ body surface area (BSA) were excluded. The primary endpoint of the study was the potential exposure of the surgical staff to cisplatin via surfaces during HITOC. For this purpose, a quantitative detection of platinum as a marker for the cytostatic drug cisplatin on defined surfaces in the OR was performed. The approval of the ethics committee of the University Regensburg was obtained (Reference number: 20-1698-101). Informed consent was waived, as the examinations do not affect the patient or change the procedure of the HITOC and only serve quality assurance and safety at work.

### HITOC

The HITOC was conducted after the resection of the pleural tumor manifestations, which was performed always with the aim of a macroscopic complete tumor resection. The thoracotomy was closed after insertion of one inflow and two to three outflow drains. After connecting the drainages to the perfusion system, the patient's thorax was evacuated by adding a priming volume of 3–4 l isotonic (0.9%) sodium chloride solution to remove air until a stable perfusion circuit was obtained. Once a temperature of 42 °C was reached at the outflow drainage, the chemotherapeutic agents were injected into the circulation as a bolus. The total volume of solution in the circulation varied depending on the individual dimensions of the thoracic cavity. The dosage of cisplatin was 175 mg/m^2^ body surface area in all patients. After completion of the 60-min HITOC, the perfusion volume was passively released and the drainages were connected to the chest drainage units. The tube and reservoir system were disposed in dedicated containers for cytostatic drugs and the perfusion device was cleaned. The patient was regularly extubated in the OR. The duration between completion of the HITOC and subsequent cleaning of the entire OR usually is about 20 min.

### Sampling sites

**Fig. 1:**
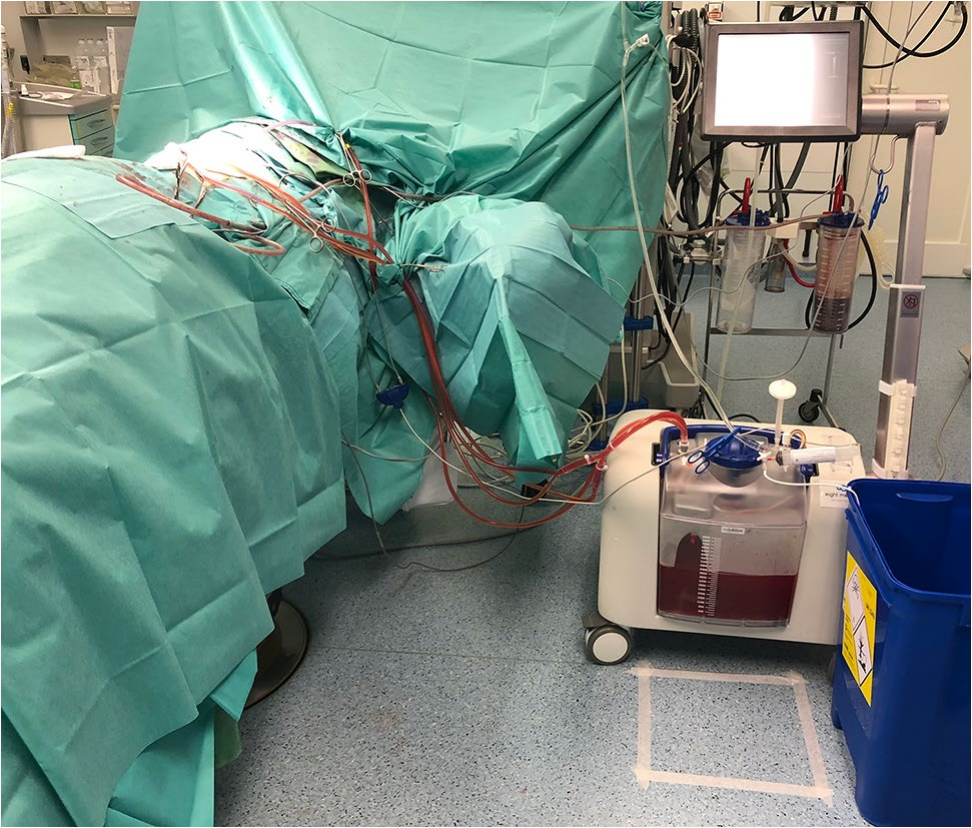
Demonstration of the perfusion setup. The picture shows the perfusion device with connected drainages and a marked area for wipe sampling in front. The cytostatic agents are injected above this area after a stable circulation has been established

**Fig. 2:**
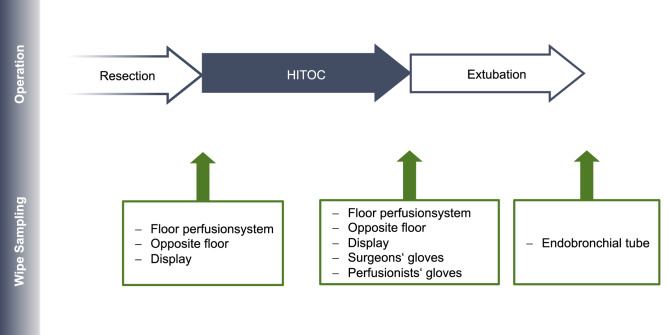
Overwiew of the sampling procedure

In total, six surface sampling areas were defined: display (touch screen) of the perfusion device (228 cm^2^), the floor area below the perfusor (900 cm^2^), the floor area below the surgeon (900 cm^2^), the inside of the breathing tube and the outside surface of both gloves (palm area, approximately 200 cm^2^) of the surgeon and the perfusionist directly after the end of the perfusion (Fig. 1). The display and the floor locations were sampled before the start of the surgery (cleaning), at the beginning of the perfusion and at the end of the perfusion to adjust for circumstantial contaminations by previous surgeries (Fig. 2). Blank samples from the gloves and the breathing tube were taken at the beginning of the study.

### Wiping procedure

The wipe sampling followed an established procedure (Schmaus et al. 2002). In detail, each surface was consecutively wiped with three circular filters papers (Quantitative Grade 391 Filter Papers, 90 mm diameter, Sartorius, Göttingen, Germany), which were folded to an easy-to-hold shape with a final wiping surface of ~ 7.5 cm^2^. Immediately prior to the sampling, the filters were moistened with 0.1% hydrochloric acid, except for the perfusor touch display, which was wiped with 0.9% sodium chloride solution. For each sampling location, a new pair of gloves was used to avoid cross-contamination. The filters for each sampling location were collected into a single screw-cap glass container, transported to the laboratory and stored at room temperature until analysis.

### Analytical procedure

Total platinum (Pt) concentrations in wipe samples were determined as a marker for cisplatin. For analysis, the wipe samples were extracted with 2% hydrochloric acid for 1 h and analyzed by voltammetry as described previously (Ensslin et al. 1994). In detail, 1 ml of the extract was transferred into a quartz vessel and 5 ml ultrapure water, 100 µl sulfuric acid (96%, Merck suprapur) and 200 µl hydrogen peroxide (30%, Merck suprapur) were added. The samples were UV-irradiated for 2 h using a MAUV-2X UV digestor (Maassen, Reutlingen, Germany) and afterwards analyzed by voltammetry for Pt determination (Methrom, Filderstadt, Germany). The quantitation was based on the standard addition method. In detail, between 10 and 100 pg of platinum (as H_2_PtCl_6_, 2.1 µg/l in 1% sulfuric acid) were added directly to the sample after initial the voltammetric analysis. Finally, platinum levels of the samples were multiplied by 1.54 to obtain cisplatin levels.

### Statistical analysis

First, the data were collected in tabular form using Excel, Version 15.0 (Microsoft Corporation, Redmond, USA). Blank values were subtracted from the results. Afterwards, statistical analysis was performed using IBM SPSS Statistics, Version 26 (IBM Corporation, Armonk, USA). All data are presented as median and interquartile range (IQR). Mean platinum levels were tested against guidance values reported in the literature using a one-sample *t* test (Schierl et al. 2009). In detail, a low contamination was defined at cisplatin levels < 0.9 pg/cm^2^, a moderate contamination at cisplatin levels between 0.9 and 6 pg/cm^2^ and a severe contamination at cisplatin levels > 6 pg/cm^2^ (Schierl et al. 2009). The differences were considered significant for a p value of less than 0.05.

## Results

**Fig. 3:**
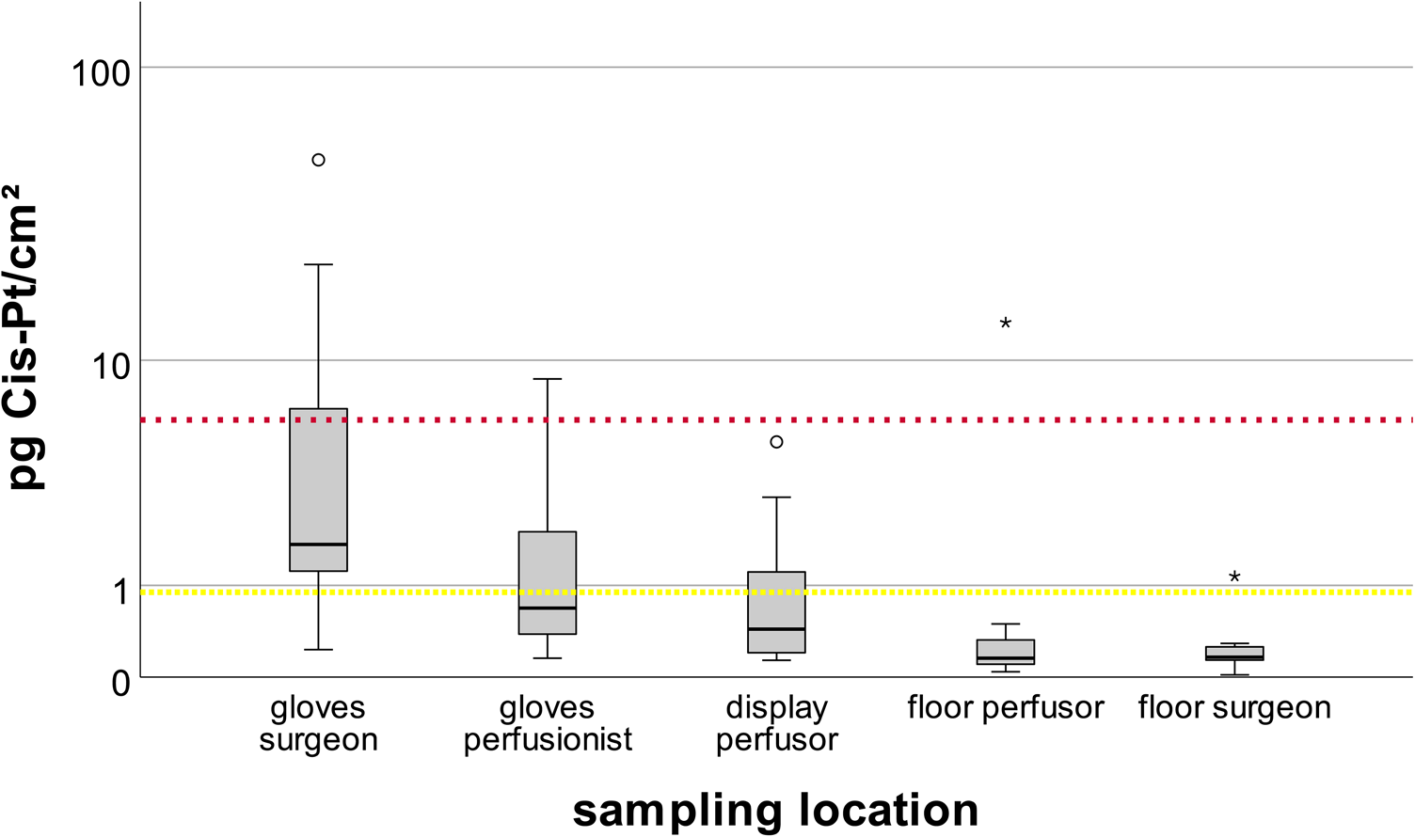
Cisplatin contamination on surfaces in the OR after HITOC. Yellow Line: 0.9 pg Cis-Pt/cm^2^ (moderate contamination). Red line: 6 pg Cis-Pt/cm^2^ (severe contamination). ∘: mild outlier (distance to quartiles between 1.5 and 3 times the interquartile range (IQR), ∗ : extreme outlier (distance to quartiles greater than 3 times the IQR (color figure online)

The results for each sampling location are given in Table 1. Corresponding box plots of the post-HITOC results are shown in Fig. 3. In the following, the individual locations of possible direct or indirect contamination in the OR are described.

**Table 1:** Summary of the wipe sampling results

Sampling location	Sample area (cm^2^)	GM	Minimum	25th percentile	Median	75th percentile	Maximum
		pg Cis-Pt/cm^2^					
Gloves surgeon^a^	200	2.50	0.23	1.02	1.73	10.38	49.06
Floor surgeon (pre)	900	0.11	0.02	0.07	0.12	0.23	0.60
Floor surgeon (post)	900	0.16	0.02	0.13	0.16	0.26	1.14
Gloves perfusionist^a^	200	0.86	0.15	0.37	0.69	2.10	8.54
Display perfusor (pre)	228	0.50	0.04	0.24	0.57	1.28	3.37
Display perfusor (post)	228	0.59	0.13	0.20	0.44	1.64	4.92
Floor perfusor (pre)	900	0.16	0.07	0.08	0.21	0.25	0.31
Floor perfusor (post)	900	0.23	0.04	0.08	0.15	0.37	13.62

Sampling location	Sample area (cm^2^)	GM	Minimum	25th percentile	Median	75th percentile	Maximum
		ng Cis-Pt					
Breathing tube (post)^a^	N.a	0.13	0.03	0.06	0.15	0.33	0.40

*GM* geometric mean, *Cis-Pt* cisplatin, *N.a* not applicable, *SD* standard deviation

^a^For single-use products, the pre-procedural value was measured once

### Gloves

The highest median levels of Cis-Pt (cisplatin) were found on the surgeon’s gloves (1.73 pg Cis-Pt/cm^2^) followed by the gloves of the perfusionist (0.69 pg Cis-Pt/cm^2^). However, the results showed a high variance. In five samples (50%) cisplatin levels on the surgeon's gloves showed a moderate contamination and four (40%) were severely contaminated (> 6 pg Cis-Pt/cm^2^). The gloves of the perfusionist showed significantly lower contamination. In fact, only three samples (30%) were moderately contaminated. In the one-sample *t* test the surface contamination of the perfusionist’s glove lies significantly below the reference value of a severe contamination (*p* < 0.001; Table 2).

**Table 2:** One sample *t* test of contamination after HITOC to reference values

	Mean ± SD	Test value = 0.9 pg Pt/cm^2^			Test value = 6 pg Pt/cm^2^		
		Mean difference	95% CI of the difference	*p* value	Mean difference	95% CI of the difference	*p* value
Gloves surgeon (pg Cis-Pt/cm^2^) (*n* = 10)	9.51 ± 15.3	8.61	− 2.3–19.6	0.109	3.51	− 7.4 to 14.5	0.486
Gloves perfusionist (pg Cis-Pt/cm^2^) (*n* = 10)	0.89 ± 0.81	− 0.01	− 0.6–0.6	0.973	− 5.11	− 5.7 to − 4.5	< 0.001*
Display (pg Cis-Pt/cm^2^) (*n* = 10)	1.16 ± 1.56	0.26	− 0.9–1.4	0.614	− 4.84	− 6 to − 3.7	< 0.001*
Floor perfusion system (pg Cis-Pt/cm^2^) (*n* = 10)	1.53 ± 4.26	0.63	− 2.4–3.7	0.651	− 4.47	− 7.5 to 1.4	0.009*
Floor surgeon (pg Cis-Pt/cm^2^) (*n* = 10)	0.26 ± 0.32	− 0.64	− 0.9 to − 0.4	< 0.001*	− 5.74	− 6 to − 5.5	< 0.001*

*Cis-Pt* cisplatin, *CI* confidence interval, *SD* standard deviation

*Statistical significance

### Perfusion device

**Fig. 4:**
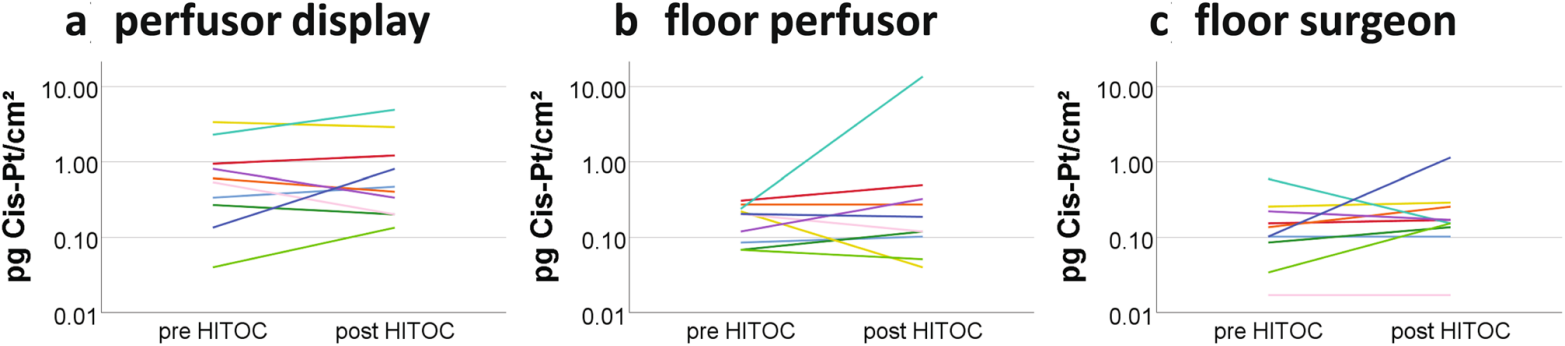
Individual cisplatin levels on the perfusor display (**a**), the floor below the perfusor (**b**) and the floor below the surgeon (**c**) before and after HITOC

Relatively high concentrations were also observed on the touch display of the perfusion device. It is worth mentioning that two moderate (20%) and one severe (10%) contaminations were observed at this location in the cleaning sample already. After HITOC, there were only three (30%) samples found in moderate contamination ranges. Comparing the values of the display before and after HITOC, no clear trend is apparent since the individual value of the sample before perfusion was sometimes even higher than after HITOC (Fig. 4). The mean cisplatin level of 1.16 pg Cis-Pt/cm^2^ on the display was significantly below the reference value of a severe contamination (*p* < 0.001) (Table 2).

### Floor

In the floor area next to the perfusion device as well as on the opposite side, platinum could be detected both before and after the HITOC. At both locations, however, observed values were low after HITOC and below the lower reference range of 0.9 pg Cis-Pt/cm^2^ in 90% of cases. In one case (10%) the cisplatin level next to the perfusion device increased from 0.24 pg/cm^2^ pre-HITOC to 13.62 pg/cm^2^ post-HITOC which represented a severe contamination. One sample (10%) of the opposite floor surface was classified as moderate contamination. The data revealed no clear trend between cleaning, pre-surgery and post-surgery samples (Fig. 4). Both floor areas had levels significantly below the reference value of a severe contamination in the one-sample *t* test (*p* = 0.009 and < 0.001).

### Endobronchial tube

After extubation, the endobronchial tube was wiped endoluminally. The tube thus contained condensed breathing air from the patient. Compared to the other investigated locations, the wiped area in the endobronchial tube was very small (~ 7.5 cm^2^). Therefore, the differences in the observed cisplatin concentration before and after HITOC consisted of minimal changes in the absolute values. A conversion to a surface concentration would disproportionately represent these minimal deviations, so that the absolute values are shown. Absolute levels of cisplatin (median: 0.15 ng Cis-Pt) in the endobronchial tube were relatively low, but higher than the blank sample (0.05 ng Cis-Pt).

## Discussion

It has been previously demonstrated that intraoperative chemotherapy methods, e.g. Hyperthermic Intraperitoneal Chemotherapy (HIPEC) and Pressurized Intraperitoneal Aerosol Chemotherapy (PIPAC), can result in substantial contamination of OR surfaces with the applied cytostatic drugs. For both surgical methods, surface contaminations were observed on the perfusion device, the floor and the gloves of surgeons and perfusionists. However, the studies showed that surgeries with overall low contaminations and occupational exposure are possible if adequate occupational safety and cleaning standards are ensured during and after these surgical procedures (Ametsbichler et al. 2018; Schierl et al. 2012). However, the contamination of surfaces in the OR's working environment during HITOC remains unclear.

In our study, cisplatin levels on individual OR surfaces showed median values of 0.12–1.73 pg Cis-Pt/cm^2^. There were some considerable outliers with values of up to 49 pg Cis-Pt/cm^2^ on the gloves of the surgeon. Also in the context of intraabdominal procedures such as HIPEC and PIPAC samples widely ranged and outliers were detected with platinum concentrations up to 110.000 pg Pt/cm^2^ (equals 169.000 pg/cm^2^ Cis-Pt) on the reservoir of the perfusion device (Schierl et al. 2012; Ametsbichler et al. 2018).

There are no threshold values for surface contaminations with cytostatic drugs. However, contaminations with carcinogenic as well as probably carcinogenic substances should be as low as possible to avoid circumstantial exposure. Most experience in this regard exists in the setting of pharmacies preparing cytostatic drugs, where contamination was detected on surfaces throughout the entire room with maximum values of 100 pg Pt/cm^2^ (Brouwers et al. 2007; Mason et al. 2005). After examining 102 German pharmacies, Schierl et al. introduced the 50th (0.6 pg Pt/cm^2^ ≙ 0.9 pg cisplatin/cm^2^) and 75th (4 pg Pt/cm^2^ ≙ 6 pg cisplatin/cm^2^) percentiles of the platinum analysis in 1008 wipe samples as guidance values to compare the results between different locations (Schierl et al. 2009).

In our study, the wipe samples of the surgeon’s gloves showed the highest level of contamination. This localization was also the only one with a median contamination level above the reference value of 0.9 pg Cis-Pt/cm^2^ (moderate contamination), all other localizations were below it. Since they are disposed of immediately after HITOC, the risk of exposure and cross-contamination in clinical practice can be presumed to be low if the gloves are disposed properly. Besides a perfusion on the closed chest performed in this study, perfusion with an open chest is also performed in some clinics internationally (Ried and Hofmann 2013). This approach is certainly likely to result in significantly higher levels of glove contamination. On average, the contaminations on the other surfaces were relatively low and showed no clear trend between cleaning, pre-surgery and post-surgery samples (Fig. 4). In some cases, the cleaning samples showed even higher levels of contamination than those before and after HITOC. On the one hand, this can be explained by the fact that the perfusion device was occasionally also used for other procedures such as HIPEC. On the other hand, this contamination may be caused by accidental cross-contamination due to the final cleaning of the perfusion device. However, after one HITOC, cisplatin levels on the display (4.92 vs 2.29 pg/cm^2^) and the floor next to the perfusion device (13.62 vs 0.24 pg/cm^2^) were clearly higher compared than in samples taken at the start of the procedure. This may be explained by an accidental spillage of the cisplatin solution during the procedure and subsequent cross-contamination. In fact, cisplatin levels of the perfusionist’s glove (2.00 pg/cm^2^) from the same day were relatively high, too. These pre-surgery and post-surgery samples in our samples should be addressed by a thorough cleaning procedure of the perfusion device after HITOC. We recommend cleaning of critical surfaces using a two-step procedure by first using an aqueous cleaning agent followed by an alcoholic solution (e.g. 70% isopropanol) directly after finishing HITOC.

The floor areas showed similar values in median after HITOC both close to the perfusion device and further away, but the variability of contamination close to the perfusion device was considerably higher. This can be explained by the fact that the cytostatics were introduced into the circulation directly above this test area, thus posing a risk of unintentional spillage of the substances. In one HITOC, cisplatin levels on the floor next to the surgeon increased from 0.10 at the start to 1.14 pg/cm^2^ after the procedure. Again, this may be caused by an accidental spillage during the perfusion or undetected leakage of surgical wounds.

We also investigated the potential contamination of the breathing tube as a potential source of airborne exposure to cisplatin. Therefore, we wiped the endobronchial tube endoluminally and thus collected condensed exhaled breath of the patient. The endobronchial tube showed cisplatin levels above the blank value, although in very small amounts. Since the measurement was taken in a distance from the distal end of the tube (> 10 cm) it can, therefore, be assumed that patients' breathing air contained cisplatin during/after HITOC. Due to its high molecular weight, cisplatin's vapor pressure is low, and so is its tendency to form aerosols (Connor et al. 2000). However, there is a possibility that cisplatin may be transported with aerosol from the respiratory tract and thus may be eventually released. The possibility of airborne exposure to cisplatin in healthcare settings has rarely been addressed in the literature (deWerk Neal et al. 1983; Ametsbichler et al. 2018). During one HITOC, we sampled ambient air of the patient after extubation for platinum analysis, but levels were below the limit of detection (data not shown). Similar results have been reported by Ametsbichler et al. for PIPAC (Ametsbichler et al. 2018). Thus, further air sampling was waived. Based on these measurements and the results of the samples on the endobronchial tube we believe that the risk of airborne particle inhalation during and after HITOC is very low and that the risk of dermal exposure is more relevant for employees. Consequently, personal protective equipment consisting of protective gloves and gowns should be used in airway management and other situations where there is a risk of direct contact with body secretions.

## Conclusion

Hyperthermic intrathoracic chemotherapy is generally safe to perform, but there are critical areas to consider. After each use, the perfusion device should be thoroughly cleaned in a two-step procedure by first using an aqueous cleaning agent followed by an alcoholic solution. The risks of direct contact with the cytostatic drug can be minimized very well by a correct use of personal protective equipment and careful handling. During and after HITOC, the protective equipment should include gloves, reinforced gowns, and safety glasses to prevent dermal uptake. Gloves should be worn for any patient contact or contact with near-patient surfaces and disposed of immediately afterwards.

### Limitations

In this study, only selected sites were monitored that the authors felt to be especially relevant for direct contact to cisplatin or as an origin of cross-contamination. No systematic ambient air study has been conducted to evaluate airborne transmission of cisplatin. The examination of the endobronchial tube in this regard represents only indirect evidence of aerosol-bound transmission. Although our results suggest that the risk of an accidental exposure of the OR personnel to cisplatin can be considered to be very low, biomonitoring would have been necessary to ultimately exclude any uptake of cisplatin.

## Data Availability

Raw data will be made available upon reasonable request.
